# Alternative Model of Personality Disorders (DSM-5) Predicts Dropout in Inpatient Psychotherapy for Patients With Personality Disorder

**DOI:** 10.3389/fpsyg.2019.00952

**Published:** 2019-04-30

**Authors:** Mareike Busmann, Johannes Wrege, Andrea H. Meyer, Franziska Ritzler, Moira Schmidlin, Undine E. Lang, Jens Gaab, Marc Walter, Sebastian Euler

**Affiliations:** ^1^Department of Psychosomatics and Psychotherapy, Psychiatric University Hospital Basel, University of Basel, Basel, Switzerland; ^2^Department of Psychology, University of Basel, Basel, Switzerland; ^3^Department of Consultation Psychiatry and Psychosomatics, University Hospital Zürich, Zurich, Switzerland

**Keywords:** Alternative Model of Personality Disorders, personality functioning, personality disorders, dropout, therapeutic alliance

## Abstract

**Objective:**

Criterion A serves as the fundamental diagnostic criterion of the Alternative Model of Personality Disorders in section III of the Diagnostic and Statistical Manual 5. Consisting of a self- and an interpersonal dimension, it defines the construct of personality functioning as a general and dimensional factor of personality disorders. This study aimed to explore criterion A along with well-established treatment dropout predictors, e.g., sociodemographic factors, personality disorder diagnosis, symptom severity, and the therapeutic alliance.

**Methods:**

The sample consisted of 132 patients diagnosed with personality disorder in a psychotherapeutic inpatient treatment. Cox proportional hazard regression models and a lasso model were applied.

**Results:**

28% of the sample prematurely discontinued treatment. The risk for dropout was 2.3 times higher for patients with high impairments in self-functioning as assessed with criterion A. Moreover, a positive therapist-rated therapeutic alliance was associated with a lower dropout risk.

**Conclusion:**

The study suggests criterion A is a useful clinical indicator by identifying patients with personality disorder with a higher risk for dropout. An individualized therapeutic approach for such patients might be required.

## Introduction

Dropout, that is, premature termination of psychotherapy, is a recurring challenge for therapists and patients in mental health services and is often interpreted as a limitation in the psychotherapeutic process. Meta-analyses suggest an overall dropout rate of roughly 20% (Swift and Greenberg, 2012, 2014; Swift et al., 2017). The cost of dropout is substantial and includes disadvantages for patients, therapists, and society. This has led to a growing interest in understanding its underlying factors (Swift and Greenberg, 2012).

Current literature suggests that dropout is a particular challenge in the treatment of patients with personality disorder (PD). For example, in a meta-analysis by Swift and Greenberg (2012), the dropout rate of patients with PD was significantly higher (25.6%) compared to patients with mood disorders (17.4%) or anxiety disorders (16.2%).

In recent years, there has been much agreement that personality functioning as a dimensional and general factor presents a valuable feature in differentiating PD from other mental disorders (Hopwood et al., 2011). Consequentially, the Alternative Model for Personality Disorders in section III of the Diagnostic and Statistical Manual 5 (DSM-5) includes as the first diagnostic criterion (criterion A) the Level of Personality Functioning Scale (LPFS). The LPFS determines a PD diagnosis in the case of a “moderate or greater impairment in personality (self/interpersonal) functioning” (Bender et al., 2011; Skodol, 2012; American Psychiatric Association, 2013, p. 775). To the best of our knowledge, little is known about the connection between the LPFS and outcome in psychotherapy.

Our study aimed to investigate the dropout rate in a naturalistic sample of patients with PD in an inpatient setting. Moreover, we evaluated personality functioning based on the criterion A of the Alternative Model of Personality Disorders (DSM-5) as a potential novel predictor for dropout along with other conventional factors, such as sociodemography, PD diagnosis, symptom severity, and therapeutic alliance from both the therapist’s and the patient’s perspectives. To the best of our knowledge, this is the first study evaluating criterion A in association with dropout.

## Materials and Methods

### Participants

The sample consisted of 132 inpatients from the Centre of Psychosomatics and Psychotherapy of the Psychiatric University Hospital Basel. All patients passed the standardized admission process including two diagnostic outpatient sessions and were thereafter admitted to the inpatient PD unit for a disorder-specific treatment. Inclusion criteria for the study were a clinical diagnosis of a PD and PD diagnosis according to the criterion A of the Alternative Model of Personality Disorders in the DSM-5 (American Psychiatric Association, 2013). Exclusion criteria were substance abuse one week before admission, psychotic symptoms, intellectual disability, and age below 18. All patients signed a written informed consent and were fully anonymized.

### Treatment

The PD-specific psychotherapeutic inpatient treatment is a highly structured and multimodal psychotherapy program combining evidence-based psychotherapeutic approaches (Sollberger et al., 2015; Euler et al., 2018). During 80 inpatient days, the treatment consisted of two weekly 45 min sessions of individual short-term psychodynamic psychotherapy (Leichsenring et al., 2004) and three weekly 75 min sessions of Mentalization-Based Group Therapy (Karterud, 2015). Therapists were advanced clinical psychologists and psychiatrists under regular weekly supervision conducted by a senior psychiatrist. Furthermore, the following therapy components are constitutional part of the program: two weekly sessions of clinical management by primary nurses, psychiatric consultation upon request, art therapy, music therapy, body work therapy, and group training in mindfulness and skills according to the Dialectical Behavioral Therapy (Linehan, 2014). Following discharge, patients continue the daily schedule for ten days after having partly re-inhabited their homes.

### Measurements

#### Criterion A

Criterion A of the Alternative Model of Personality Disorders is assessed by the LPFS. The LPFS evaluates the severity of impairments in core personality functions (Bender et al., 2011; American Psychiatric Association, 2013). It consists of two dimensions with two scales each, the self-functioning (identity and self-direction) and interpersonal-functioning (empathy and intimacy), as well as a global scale. The clinician determines a patients’ functioning in the respective area from 0 to 4 (little or no, some, moderate, severe, extreme impairment). A higher score indicates a higher level of impairment and the diagnosis of a PD requires at least a moderate level on the global scale score. According to Morey et al. (2013), we used at least a moderate impairment in the global personality functioning for defining a PD level which is described as reliable. In the current study, criterion A was assessed by the senior psychiatrist of the treatment after a psychodynamic clinical interview that is comparable to the interview according to the Operationalized Psychodynamic Diagnosis (OPD Task Force, 2008) using in the study of Zimmermann et al. (2014). Thus, our procedure can be accepted as reliable. Reliability and validity of the LPFS have been confirmed (Bender et al., 2011; Few et al., 2013; Morey et al., 2013; Zimmermann et al., 2014). In our sample, the internal consistency was acceptable for both dimensions considering that each dimension consisted of two items (self-functioning: Cronbach’s α = 0.66; interpersonal-functioning: Cronbach’s α = 0.63).

#### Personality Disorder Diagnosis

The Structured Clinical Interview for the Diagnostic and Statistical Manual of Mental Disorders II (SCID-II) assesses the primary diagnosis of a PD (Gibbon et al., 1997). In the current study, advanced clinical psychologists or psychiatrists administered the interviews. To ensure the reliability of the ratings, consensus deliberations were regularly held and supervised by senior researchers. The reliability of the SCID-II has been shown in several studies (Lobbestael et al., 2011). For our statistical analysis, we used the number of fulfilled criteria within each PD diagnosis.

#### Clinical Global Impression – Severity Scale

The Clinical Global Impression-Severity Scale (CGI-S) measures the severity of mental illness (Guy, 1976). The clinician estimated one item on a seven-point Likert scale from 1 (normal, not at all ill) to 7 (among the most extremely ill patients). The internal consistency is given (Leon et al., 1993).

#### Global Assessment of Functioning

The Global Assessment of Functioning (GAF) assesses the level of psychological functioning (Ramirez et al., 2008). The clinician ranked the patient on one item including a ten-step scale from 1 (severely impaired) to 100 (extremely high functioning). The reliability is good (Hay et al., 2003).

#### Brief Symptom Checklist

The Brief Symptom Checklist (BSCL) investigates psychological strain (Franke, 2016). The self-report questionnaire consists of nine scales containing the dimensions anger, hostility, anxiety, depression, paranoid ideation, phobic anxiety, psychoticism, somatization, interpersonal sensitivity, and obsessive-compulsive. The patient rated each of the 53 items on a five-point Likert scale from 0 (not at all) to 4 (extremely). A global severity index (GSI) was computed and showed a high internal consistency (Franke, 2016). In our sample, the internal consistency of the GSI was also high (Cronbach’s α = 0.96).

#### Inventory of Personality Organization

The Inventory of Personality Organization (IPO) captures the level of psychopathological personality structure (Lenzenweger et al., 2001). The short version includes 16 items and the patient rated each item on a six-point Likert scale from 1 (never true) to 5 (always true). A global mean score was determined. The IPO has a high internal consistency (Zimmermann et al., 2013). Similarly, the internal consistency was high in our sample (Cronbach’s α = 0.85).

#### Scale to Assess Therapeutic Relationships

The Scale to Assess Therapeutic Relationships (STAR) was especially developed to investigate the therapeutic alliance between patient and it’s therapist in individual therapy (McGuire-Snieckus et al., 2007; Gairing et al., 2011). The STAR exists in two versions. While one version refers to the perspective of the patient (STAR-P), the other version describes the view of the clinician (STAR-C). The STAR-P consists of three subscales, namely positive collaboration, positive clinician input, and non-supportive clinician input. The STAR-C includes three subscales called positive collaboration, positive clinician input, and emotional difficulties. The patient and the individual therapist ranked each item of their version on a five-point Likert scale from 0 (never) to 4 (always). The non-supportive clinician input and the emotional difficulties scales were recoded. Afterward, a mean score for each scale and an overall mean score for each version were calculated. According to Gairing et al. (2011), the internal consistency of the overall scores is high which was also reflected in our sample (STAR-P: Cronbach’s α = 0.90; STAR-C: Cronbach’s α = 0.88).

### Operationalization of Dropout

The sample was divided into two groups based on the outlined treatment structure: completers and non-completers. By definition, completers completed the inpatient treatment including day 80, whereas non-completers dropped out before. Thereby, dropout is defined by a full exit of the hospital.

### Study Protocol

All study days were integrated in the treatment process (days 1–80). Informed consent, SCID I and II, demographic variables, symptom severity questionnaires (CGI, GAF, BSCL, IPO), and the assessment of the criterion A were administered during the first week (day 1–7). The STAR regarded only the individual therapy, therefore, was evaluated by the individual therapist and its respective patient during four periods (day 7–15, 25–35, 50–60, 80–90). For the statistical analysis, we used the last STAR measurement closest to dropout for the non-completers group, and the last one before the regular completion of the treatment for the completers group.

### Statistical Analysis

Univariate Cox proportional hazard models were used to examine the individual impact of each predictor on dropout. We assessed the criterion A (LPFS), sociodemographic variables (age, gender, education years), diagnosis of PD (SCID-II), symptom severity (CGI, GAF, BSCL, IPO), and the therapeutic alliance (STAR) on the probability of dropout. The proportional hazards condition was met. A significance threshold of *p* < 0.05 was set for all univariate analyses. Further, a multivariate model was used to test each predictor’s impact on dropout adjusted for all other predictors in the model. In order to avoid model overfitting due to the high number of predictors relative to the number of dropouts leading to a low predictive accuracy, we used a variable selection procedure, the least absolute shrinkage and selection operator lasso method. Since the outcome variable was time to events, the lasso was based on a Cox model. At last, we correlated the predictors. Univariate Cox models were run with the Statistical Package for Social Sciences (SPSS 24), while the multivariate lasso model was run with R (R 3.3.0) including the R package glmnet (Friedman et al., 2010).

## Results

### Sociodemographic and Clinical Data

Sociodemographic and clinical data of both groups are illustrated in Table 1.

**Table 1:** Sociodemographic and clinical data (*N* = 132).

	Completers (*n* = 95)	Non-completers (*n* = 37)
	*M* (*SD*)/*n* (%)	*M* (*SD*)/*n* (%)
**Demographics**		
Age in years	29.7 (9.5)	28.5 (8.6)
Female	56 (58.9)	26 (70.3)
Male	39 (41.1)	11 (29.7)
Education in years	13.6 (2.6)	12.9 (2.9)
Employed	27 (28.4)	4 (10.8)
Unemployed	68 (71.6)	33 (89.2)
Single	63 (66.3)	26 (70.3)
Marriage, partnership	32 (33.7)	11 (29.7)
**Criterion A (DSM-5)**		
Global personality functioning	2.8 (0.6)	3.0 (0.7)
Self-functioning	2.6 (0.6)	3.0 (0.5)
Identity	2.7 (0.7)	3.0 (0.7)
Self-direction	2.6 (0.7)	2.9 (0.6)
Interpersonal-functioning	2.6 (0.6)	2.7 (0.7)
Empathy	2.5 (0.8)	2.5 (0.9)
Intimacy	2.6 (0.7)	2.8 (0.7)
**Axis II Disorder (DSM-IV)**		
Paranoid	9 (9.5)	3 (8.1)
Schizoid	7 (7.4)	1 (2.7)
Schizotypal	2 (2.1)	2 (5.4)
Antisocial	6 (6.3)	5 (13.9)
Borderline	60 (63.2)	28 (75.7)
Histrionic	4 (4.2)	1 (2.7)
Narcisstic	12 (12.6)	4 (10.8)
Avoidant	21 (22.1)	9 (24.3)
Dependent	9 (9.5)	3 (8.1)
Obsessive-compulsive	10 (10.5)	5 (13.5)
Depressive	11 (11.6)	3 (8.1)
Negativistic	15 (15.8)	5 (13.5)
Amount	1.8 (1.6)	1.9 (1.6)
**Axis I Disorder (DSM-IV)**		
Depression	32 (33.7)	13 (35.1)
Anxiety disorders	22 (23.2)	5 (13.5)
Eating disorders	11 (11.6)	7 (18.9)
Substance disorders	45 (47.4)	10 (27.0)
Somatoform disorders	4 (4.2)	0 (0)
Posttraumatic stress disorder	5 (5.3)	2 (5.4)
**Symptom Severity**		
Clinical Global Impression-Severity Scale	5.0 (0.5)	5.1 (0.7)
Global Assessment of Functioning	42.0 (7.2)	43.5 (9.0)
Brief Symptom Checklist-Global Severity Index	1.3 (0.6)	1.4 (0.7)
Inventory of Personality Organization	2.5 (0.6)	2.3 (0.6)
Medication	63 (66.3)	28 (75.7)

*Completers ≥ 80 treatment days, non-completers < 80 treatment days, DSM = Diagnostic and Statistical Manual of Mental Disorders.*

### Dropout Rate

Overall, 28% of all patients prematurely discontinued psychotherapy. Completers (*n* = 95, 72.0%) obtained 89.5 treatment days (*SD* = 1.1, min = 85, max = 90), non-completers (*n* = 37, 28.0%) obtained 52.5 treatment days (*SD* = 20.2, min = 13, max = 79) on average.

### Predictors of Dropout

The impact of all investigated predictors on dropout based on the univariate cox models is presented in Table 2.

**Table 2:** Predictors of dropout (*N* = 132).

				Goodness-of-fit statistic		
	HR	CI 95%	*p*	Chi-Quadrat	df	*p*
**Criterion A (DSM-5)**						
Global personality functioning	1.47	0.86–2.54	0.16	1.96	1	0.16
Self-functioning	2.32	1.30–4.13	<0.01^∗∗^	8.02	1	0.01
Identity	1.97	1.17–3.31	0.01^∗^	6.53	1	0.01
Self-direction	1.81	1.11–2.96	0.02^∗^	5.67	1	0.02
Interpersonal-functioning	1.26	0.76–2.08	0.37	0.80	1	0.37
Empathy	1.01	0.67–1.54	0.95	0.01	1	0.95
Intimacy	1.41	0.91–2.32	0.13	2.33	1	0.13
**Personality Disorder (DSM-IV)**						
Paranoid	0.99	0.81–1.22	0.95	0.00	1	0.95
Schizoid	0.85	0.61–1.18	0.33	0.98	1	0.32
Schizotypal	1.03	0.81–1.31	0.83	0.05	1	0.83
Antisocial	1.05	0.84–1.32	0.65	0.20	1	0.65
Borderline	1.11	0.96–1.28	0.16	2.03	1	0.16
Histrionic	1.12	0.92—-1.35	0.25	1.33	1	0.25
Narcissistic	0.94	0.78–1.13	0.50	0.45	1	0.50
Avoidant	1.03	0.89–1.19	0.73	0.12	1	0.73
Dependent	1.00	0.84–1.18	0.97	0.00	1	0.97
Obsessive-compulsive	1.11	0.94–1.32	0.21	1.57	1	0.21
Depressive	1.00	0.85–1.18	0.97	0.00	1	0.97
Negativistic	0.94	0.77–1.14	0.51	0.43	1	0.51
**Symptom Severity**						
Clinical Global Impression-Severity Scale	1.28	0.71–2.29	0.40	0.68	1	0.41
Global Assessment of Functioning	1.02	0.98–1.06	0.40	0.70	1	0.40
Brief Symptom Checklist-Global Severity Index	1.26	0.75–2.11	0.38	0.77	1	0.38
Inventory of Personality Organization	0.66	0.38–1.14	0.14	2.21	1	0.14
**Therapeutic Alliance**						
Patient-rated overall	0.71	0.46–1.11	0.13	2.28	1	0.13
Patient-rated positive clinician input	0.69	0.47–1.01	0.05	3.73	1	0.05
Patient-rated positive collaboration	0.77	0.52–1.12	0.17	1.93	1	0.17
Patient-rated non-supportive clinician input	0.87	0.57–1.33	0.87	0.42	1	0.52
Therapist-rated overall	0.31	0.14–0.69	<0.01^∗∗^	8.37	1	<0.01
Therapist-rated positive clinician input	0.47	0.23–0.96	0.04^∗^	4.28	1	0.04
Therapist-rated positive collaboration	0.43	0.22–0.86	0.02^∗^	5.87	1	0.02
Therapist-rated emotional difficulties	0.50	0.28–0.87	0.01^∗^	6.07	1	0.01
**Sociodemographics**						
Age	0.99	0.95–1.03	0.56	0.34	1	0.56
Gender	0.64	0.32–1.30	0.22	1.56	1	0.21
Education	0.92	0.81–1.05	0.22	1.52	1	0.22

*HR = Hazard Ratio, CI = Confidence Interval, DSM = Diagnostic and Statistical Manual of Mental Disorders, HR are based on univariate Cox proportional hazard models, ^∗^p < 0.05, ^∗∗^p < 0.01.*

**FIGURE 1:**
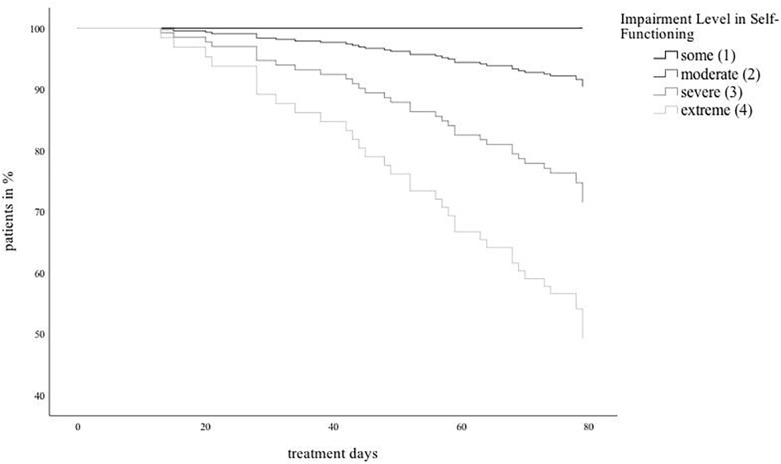
Cox curves of the sample devided in four groups according to the level of self-functioning.

Both subscales of self-functioning, identity and self-direction, significantly predicted dropout. Figure 1 illustrates the Cox curves of the sample divided in four groups according to the level of self-functioning.

Furthermore, the therapist-rated therapeutic alliance significantly predicted dropout, including all subscales. None of the other factors had a significant impact on dropout.

The analysis of the multivariate lasso model identified both self-functioning scales, identity (HR = 1.10) and self-direction (HR = 1.02), as well as the scales positive collaboration (HR = 0.93) and emotional difficulties (HR = 0.95) from the therapist’s perspective (STAR) as being predictive of dropout. The cross-validated mean squared error of the lasso model was 11.4. Thus, the multivariate lasso model verified the results of the univariate Cox models.

The correlations showed significant relations between the four LPFS dimensions: identity was significantly correlated with self-direction (*r* = 0.50, *p* < 0.01), empathy (*r* = 0.27, *p* < 0.01), and intimacy (*r* = 0.39, *p* < 0.01). Self-direction was significantly associated with empathy (*r* = 0.38, *p* < 0.01) and intimacy (*r* = 0.41, *p* < 0.01) as well as there was a correlation between empathy and intimacy (*r* = 0.46, *p* < 0.01). Moreover, there were significant correlations between identity and paranoid personality disorder (*r* = 0.19, *p* = 0.03), borderline personality disorder (*r* = 0.21, *p* = 0.02), avoidant personality disorder (*r* = 0.23, *p* < 0.01), and dependent personality disorders (*r* = 0.18, *p* = 0.04) as well as between identity and therapist-rated emotional difficulties (*r* = −0.18, *p* = 0.04), and education (*r* = −0.31, *p* < 0.01). Self-direction was significantly associated with borderline personality disorder (*r* = 0.31, *p* < 0.01), avoidant personality disorder (*r* = 0.22, *p* = 0.01), BSCL (*r* = 0.19, *p* = 0.03), IPO (*r* = 0.23, *p* < 0.01), therapist-rated emotional difficulties (*r* = −0.19, *p* = 0.03), and education (*r* = −0.33, *p* < 0.01). Empathy was significantly correlated with schizotypal personality disorder (*r* = 0.20, *p* = 0.02), borderline personality disorder (*r* = 0.19, *p* = 0.03), and negativistic personality disorder (*r* = 0.22, *p* = 0.01). Intimacy showed significant relations to CGI (*r* = 0.22, *p* = 0.01), BSCL (*r* = 0.18, *p* = 0.04), and IPO (*r* = 0.71, *p* < 0.05).

## Discussion

Our study aimed to investigate criterion A of the Alternative Model for Personality Disorders in DSM-5 as potential novel predictor of dropout in comparison with sociodemographic factors, symptom severity, PD diagnosis, and the patient- and therapist-rated therapeutic alliance. A higher impairment in self-functioning as measured by criterion A and a weaker therapist-rated therapeutic alliance were associated with higher dropout rates when controlling for all other factors.

The dropout rate of 28% of our sample was similar to studies with comparable clinical samples. For instance, Karterud (2015) obtained a dropout rate of 24% including 81% patients with at least one DSM-IV PD diagnosis.

Our most central finding was the association between low self-functioning (criterion A) and dropout. Thus, our study has shown, that, for example, the lack of boundaries between self and others, an instable self-esteem, and a disturbed emotion regulation (identity) as well as an inability to pursuit long- and short-term goals, and an inability for self-reflection (self-functioning) may lead to dropout. In consequence, it may be argued that a certain level of self-functioning is a prerequisite for structured inpatient psychotherapy for patients with PD. All evidence-based therapies for PD provide specific concepts to deal with low self-functioning; for instance, emotion regulation skills in Dialectical Behavioral Therapy or supporting self-agency in Mentalization-Based Therapy (Linehan and Wilks, 2015; Bateman and Fonagy, 2016). Our study suggests that a specific emphasis on the self-functioning features according to the underlying treatment manuals might be promising to keep patients in treatment. This may mean that it may be helpful to assess criterion A at the beginning of treatment and, in case of a higher impairment, it should be actively prioritized by therapists during the therapy sessions. Some studies have already shown that patients with PD might rather benefit from an individually tailored treatment approach (Vermote et al., 2011; Links et al., 2017).

Nevertheless, it is to observe, that the current research state of studies concerning criterion A and outcome measurements is very low. In a study with related constructs, Rubin et al. (2016) evaluated predictors of dropout in a psychodynamic treatment for patients with an Axis-I diagnosis. Utilizing a comparable scale assessing intrapsychic functioning as rated by the therapist, the authors suggested an association between lower functioning level and dropout. The result underlines its significance for diagnosis and treatment of patients with PD, but more research is needed. Interestingly, the interpersonal dimension of criterion A did not have an impact on the dropout in the current study. That is contrary to the expectations because of the particular relevance of interpersonal functions in psychotherapy of PD (Clarkin et al., 2010). Further research is needed to confirm this finding.

The therapist-rated therapeutic alliance was the second significant predictor of dropout in the current study. Thus, in addition to the self-functioning level, we might conclude that the therapist’s perception of the therapeutic alliance could be important to prevent dropout in patients with PD. Bender (2005) has already described that patients with PD have very difficult interpersonal patterns including repeating strains and ruptures that are influencing the patient-therapist-alliance in psychotherapy and may leading to dropout. In Tufekcioglu et al. (2013), the authors have shown that alliance ruptures occur more often in the therapeutic alliance of patients with PD than in patients with non-PD. In order to prevent dropout, the therapist’s perception might be very attentive to signals of a negative alliance, e.g., alliance ruptures. Meanwhile, all evidence-based treatments for PD conceptualize the issue of alliance-building as one of the major treatment targets (Bateman et al., 2015). For instance, recommendations refer to the implementation of epistemic trust, the therapist’s responsiveness, and the handling of rupture situations (Fonagy and Allison, 2014; McMain et al., 2015).

In addition, the association between dropout and the patient-rated therapeutic alliance has also been shown in previous PD studies (Lingiardi et al., 2005; Wnuk et al., 2013). Contrary to those, we found no association between the patient-rated alliance and dropout.

The sociodemographic factors, the PD diagnoses, and the symptom severity did not predict dropout. This is in contrast to other dropout studies including patients with PD (Karterud et al., 2003; Ogrodniczuk et al., 2008; Gamache et al., 2018). These partly contradictory findings have to be investigated in future studies.

Due to the naturalistic study design of the current study, we applied a quasi-experimental method. Thereby, on the one hand, we cannot control for all confounding variables leading to a low internal validity. On the other hand, the naturalistic setting includes a large homogenous clinical sample leading to a high external validity applicable to other inpatient settings. Further, it has to be considered that 67% of the patients of the current study showed a diagnosis of a borderline personality disorder according to the SCID-II. In comparison to other inpatient PD samples, this a high rate and thus limits the validity of our results for other PDs (Bender et al., 2001). Nevertheless, the sample is very homogenous and the evaluation of a more heterogenous sample consisting of patients with and without personality disorders representing the whole range of possible LPFS scores could be of great interest for further research. At last, it is important to note that the dropout rate is also influenced by the dropout definition which differs across studies (Swift and Greenberg, 2012). The dropout operationalization used in the current study was aligned with existing definitions for inpatient treatments (Kröger et al., 2014).

By showing that low self-functioning as measured with criterion A is a significant predictor of dropout in PD, this study suggests that an initial treatment focus on self-functioning according to treatment manuals of current evidence-based therapies may be required for patients with low self-functioning. The result confirms the clinical significance of criterion A as general and dimensional PD factor and its utility in clinical settings. However, its eligibility has to be confirmed for different treatment settings in future studies. Moreover, our results suggest that the therapist’s perception of an impaired alliance might serve as an important signal to introduce specific interventions in order to prevent dropout.

## Ethics Statement

The study is approved by the Ethics Committee of Northwestern/Central Switzerland (2914-078, PB_2017-00645).

## Author Contributions

SE, JW, and MW designed and directed the project. SE and MB developed the issue. MW supervised the project. MB, FR, and MS collected and processed the data. MB and AM analyzed the data. MB wrote the manuscript. AM wrote parts of the statistical analyses. SE, MW, UL, and JG assisted the writing process of the manuscript. All authors discussed the results and contributed to the final manuscript.

## Conflict of Interest Statement

The authors declare that the research was conducted in the absence of any commercial or financial relationships that could be construed as a potential conflict of interest.
